# District of Columbia

**Published:** 1896-04

**Authors:** 


### DISTRICT OF COLUMBIA.

The bill pending before Congress to establish a board of registration and examiners for the District is reported to be in a favorable position on the calendar and quite likely to become a law. We sincerely trust our fellow-veterinarians may be successful in their efforts, for nothing will strengthen the hands of those seeking to elevate the course of instruction in our schools more than these laws, well prepared and enforced.

